# Nitrogen Fertilizer Regulated Grain Storage Protein Synthesis and Reduced Chalkiness of Rice Under Actual Field Warming

**DOI:** 10.3389/fpls.2021.715436

**Published:** 2021-08-30

**Authors:** Xueqin Wang, Kailu Wang, Tongyang Yin, Yufei Zhao, Wenzhe Liu, Yingying Shen, Yanfeng Ding, She Tang

**Affiliations:** ^1^College of Agronomy, Nanjing Agricultural University, Nanjing, China; ^2^Jiangsu Collaborative Innovation Center for Modern Crop Production, Nanjing, China

**Keywords:** rice, actual field warming, nitrogen fertilizer, grain storage protein, quality, chalkiness

## Abstract

Our previous study has shown that nitrogen plays an important role in dealing with significantly increased chalkiness caused by elevated temperature. However, the role of nitrogen metabolites has not been given sufficient attention, and its regulatory mechanism is not clear. This study investigated the effects of high temperature and nitrogen fertilizer on the synthesis of grain storage protein and further explored the quality mechanism under the actual scenario of field warming. Results showed that increased temperature and nitrogen fertilizer could affect the activities of nitrogen metabolism enzymes, namely, glutamate synthetase, glutamine synthetase, glutamic pyruvic transaminase, and glutamic oxaloacetic transaminase, and the expressions of storage protein synthesis factor genes, namely, *GluA* and *GluB*, and subfamily genes, namely, *pro14, BiP1*, and *PDIL1*, which co-induced the changes of storage protein synthesis in rice grains. Furthermore, the increased temperature changed the balance of grain storage substances which may lead to the significantly increased chalky rate (197.67%) and chalkiness (532.92%). Moreover, there was a significant negative correlation between prolamin content and chalkiness, indicating that nitrogen fertilizer might regulate the formation of chalkiness by affecting the synthesis of prolamin. Results suggested that nitrogen application could regulate the related core factors involved in nitrogen metabolism pathways, which, in turn, affects the changes in the storage protein components in the grain and further affects quality. Therefore, as a conventional cultivation measure, nitrogen application would have a certain value in future rice production in response to climate warming.

## Introduction

With the improvement of living standards of people, the demand for high-quality rice is increasing. However, rice quality is extremely sensitive to temperature. With the intensification of global warming, the increase of chalkiness in rice quality traits has become a key issue that needs to be focused on. Studies conducted by artificial climate room (Xu et al., 2020) and actual field warming experiments (Rehmani et al., 2014) illustrated that high temperature was conducive to the occurrence of chalkiness (Mitsui et al., 2013). Increased grain chalkiness affects not only appearance quality but also milling and cooking quality (Cheng et al., 2005; Guo et al., 2011). Based on our previous field trials, a small scale of temperature increase during the rice grain-filling stage can lead to a significant increase in chalkiness, which will bring new challenges to the production of high-quality rice in the future and reasonable cultivation measures that are urgently needed. Nitrogen application is a simple agronomic measure, which has been proved that it can alleviate high-temperature damage through decelerating the early grain-filling rate of rice and could further reduce the occurrence of chalkiness (Dou et al., 2017; Tang et al., 2019). Although the regulatory effect of nitrogen on the physicochemical properties of rice starch had been preliminarily clarified in our previous study, the characteristics of its effects on grain nitrogen metabolism have not yet been investigated.

The formation of grain chalkiness is closely related to carbon and nitrogen metabolism. Previous studies have shown that genes contributed to the formation of chalkiness are all related to the carbon and nitrogen metabolism key enzymes (Kang et al., 2005; Fujita et al., 2007; Wang et al., 2008). Our previous studies showed that nitrogen fertilizer effectively influenced the accumulation and structural characteristics of starch, which further alleviated the rice quality under increased temperature (Tang et al., 2019). The application of nitrogen fertilizer affected the structure of rice starch, which further changed its functional properties and eventually led to the changes in the grain quality (Zhou et al., 2020). Being the second largest storage components in rice grain, rice seed storage protein (SSP) accounts for about 8–10% of grain weight (Kawakatsu and Takaiwa, 2010). The grain protein content has been proved to be significantly negatively correlated with appearance, and increased temperature could regulate the grain protein synthesis, resulting in the changes in balance of storage substance, which could further regulate the formation of grain chalkiness (Tang et al., 2018; Liu et al., 2020). Studies also showed that reasonable nitrogen application could alleviate chalkiness caused by elevated temperature (Wada et al., 2019). Therefore, understanding the mechanisms of nitrogen fertilizer on grain storage protein synthesis and appearance quality under open-field warming condition would be contributed to the establishment of reasonable cultivation measures.

Our previous studies on temperature increase and nitrogen application have clarified the effect of carbon metabolism on quality and chalkiness, but the effect of nitrogen metabolism on the formation of chalkiness is still unclear. As a simple measure suitable for actual field operations, nitrogen fertilizer can alleviate the deterioration of quality caused by climate warming, including reducing chalkiness, but its mechanism has not been fully revealed. Therefore, the purpose of this study was to explore the contribution of nitrogen metabolism to the formation of chalkiness through the application of nitrogen fertilizer under the increased open-air temperature experiment. By clarifying the regulatory effects of nitrogen application on nitrogen metabolism-related enzymes and regulatory factors under increased temperature, the results would help supplement its effect on grain nitrogen metabolites. Based on this, the mechanism of nitrogen fertilizer measures in alleviating the deterioration of rice quality could be further revealed, and this may help systematically assess the potential role of nitrogen in combating the deterioration of rice quality under climate warming.

## Materials and Methods

### Experimental Conditions

The actual field warming scene is located in the middle and lower reaches of the Yangtze River Basin, and the experiments were conducted at the Rice Research Station of Nanjing Agricultural University (31°56′39″N, 118°59′13″E, 80 m altitude). Rice was sowed on May 27, transplanted on June 15, and headed on August 26 in 2019. The experimental site soil type is clay loam, with a pH value of 6.41. The total nitrogen, available nitrogen, total phosphorus, available phosphorus, and available potassium from 0 to 20 cm soil were 1.4 g kg^−1^, 7.8 mg kg^−1^, 0.6 g kg^−1^, 20 mg kg^−1^, and 91.7 mg kg^−1^, respectively.

### Plant Materials and Treatment

The test material was the conventional *japonica* rice variety Wuyujing 3 (W3), which belongs to the high-quality *japonica* rice variety widely planted in the local area, and its chalkiness is sensitive to external temperature. The cultivation process including artificial transplanting was carried out after the nursery, and field management besides pest control was conducted on the basis of local high-yield and high-quality cultivating measures. The experimental treatments of elevated temperature and nitrogen fertilizer application are as follows: ambient temperature (CK), elevated temperature (ET), application of nitrogen fertilizer under normal temperature (CKN), and application of nitrogen fertilizer under elevated temperature (ETN). Field warming treatment was conducted through the free-air temperature enhancement (FATE) facility (Supplementary Figures 1, 2). The infrared ceramic heaters of the FATE system were used to warm the rice plants during day and night after anthesis. The detailed parameters of the system can be retrieved in our previous studies (Rehmani et al., 2014; Tang et al., 2019). A temperature and hygrometer HOBO U23-001 was placed in the rice canopy to record the temperature during grain filling, and HOBOwarePro software (Onset Computer Co., Bourne, MA, USA) was used for data processing. The total nitrogen application of CK and ET was 300 kg·hm^−2^, and the ratio of basal fertilizer, tiller fertilizer, and panicle fertilizer was 4:2:4. Compared with CK and ET, CKN and ETN with increased nitrogen fertilizer were applied with 60 kg N·hm^−2^ nitrogen at the beginning of temperature increasing treatment.

### Determination of Yield and Quality

The effective panicles of dozens of holes were randomly investigated in the no sampling area at the maturity stage, and the spikelets per panicle and seed setting rate were calculated, with three replicates in each group. Panicle samples were threshed at 70°C and dried to constant weight, and the grain weight was weighed to calculate the theoretical yield of rice. Determinations of grain quality traits were conducted (refer to our previous test procedures) (Dou et al., 2017; Tang et al., 2019), and the brief protocols are as follows: the ratio of grain length to width was measured by using a vernier caliper. The chalkiness degree was calculated by the multiplication of the chalkiness rate and chalkiness area. JLGJ 4.5 shelling machine of Taizhou Grain Industry Instrument Company was used for shelling, and the brown rice rate was calculated. Brown rice was weighed and milled by JNMJ3 rice mill for 90 s to determine the milled rice rate. Samples with integrity >80% were picked from the milled rice and weighed to calculate the head rice rate. Rapid-visco-analyzer (RVA) characteristics of rice were determined by RVA-4500, a rapid viscosity analyzer developed by Newport Scientific Instrument Company in Australia. A total of 3.00 g of rice flour with a moisture content of about 14.0% was added to the aluminum box, with 25 ml of distilled water, and quickly stir it up and down with a stirrer for 10 times to make the rice flour disperse evenly according to the AACC standard (2012). The prepared samples were tested on RVA-4500 according to the set procedure.

### Transmission Electron Microscope Observation

The spikelets were obtained from the first branch in the middle of the panicle at 6, 9, 12, 15, and 20 days after anthesis (DAA). Transverse segments (1–2 mm thick) from the same middle of the kernels were obtained to observe the morphological and structural changes and the shape and spatial arrangement of amyloplasts and protein bodies (PBs) according to the JEM-1200EX transmission electron microscope (Tang et al., 2018).

### Determination of Grain Storage Material Contents

The polarimetric method was employed to determine the total starch content of rice flour samples after 100 mesh screening. The amylose content was measured according to the standard of the People's Republic of China GB/17891-1999 high-quality rice. The grain storage proteins, namely, albumin, globulin, prolamin, and glutelin, were extracted from distilled water, dilute hydrochloric acid, ethanol, and dilute alkali. Among them, albumin, globulin, and prolamin were determined by Coomassie brilliant blue colorimetry, and glutelin was determined by biuret colorimetry. The content of 17 amino acids in the protein was detected by using the Hitachi L-8900 amino acid analyzer (Hitachi Corp, Japan) according to the hydrochloric acid hydrolysis method.

### Analysis of Enzyme Activities Related to Protein Synthesis

The spikelets collected from 6, 9, 12, 15, 20, 25, and 35 DAA were grounded into powder in liquid nitrogen. The glutamine synthetase (GS) and glutamate synthetase (GOGAT) activities were determined by using the protocols described in our previous study by Tang et al. (2018), and the glutamic oxaloacetic transaminase (GOT) and glutamic pyruvic transaminase (GPT) activities were analyzed according to the protocols described by Wu et al. (1998).

### RNA Extraction and RT-PCR

Total RNAs of test samples were extracted and purified from shelled grains of 6, 9, 12, 15, and 20 DAA according to the instructions of the RNAprep Pure Plant Kit (TIANGEN, Beijing, https://www.tiangen.com/). After extraction, the concentration and purity of total RNA were analyzed using the NanoDrop One C Ultra-micro spectrophotometer (Thermo Fisher Scientific, USA). Reverse transcription was performed using the Takara's PrimeScript™ RT Kit (Takara Biotechnology, Tokyo, Japan). The real-time quantitative analysis was conducted based on the Biosystems 7300 and StepOnePlus™ real-time PCR system. The cycling parameters were as follows: 30 s at 95°C, 40 cycles of 5 s at 95°C, and 31 s at 65°C. *Actin* was used to calculate the relative expression level of target genes. Primers used in this study are listed in Supplementary Table 1.

### Data Analysis

Data sorting and analysis were performed using Microsoft Excel 2019 (Microsoft Corporation, WA, USA) and SPSS20.0 statistical software (IBM SPSS® Statistics, NY, USA) statistical software. Origin 8.1 (OriginLab Corporation, MA, USA) was employed for figure preparation. ANOVA was used to analyze data according to a completely random design, and the averages were compared by using the Duncan's multiple range test (DMRT) based on the least significant difference test at the 5% probability level.

## Results and Analysis

### Effects of Nitrogen Fertilizer on Yield and Quality of Rice Under Elevated Temperature

**Figure 1:**
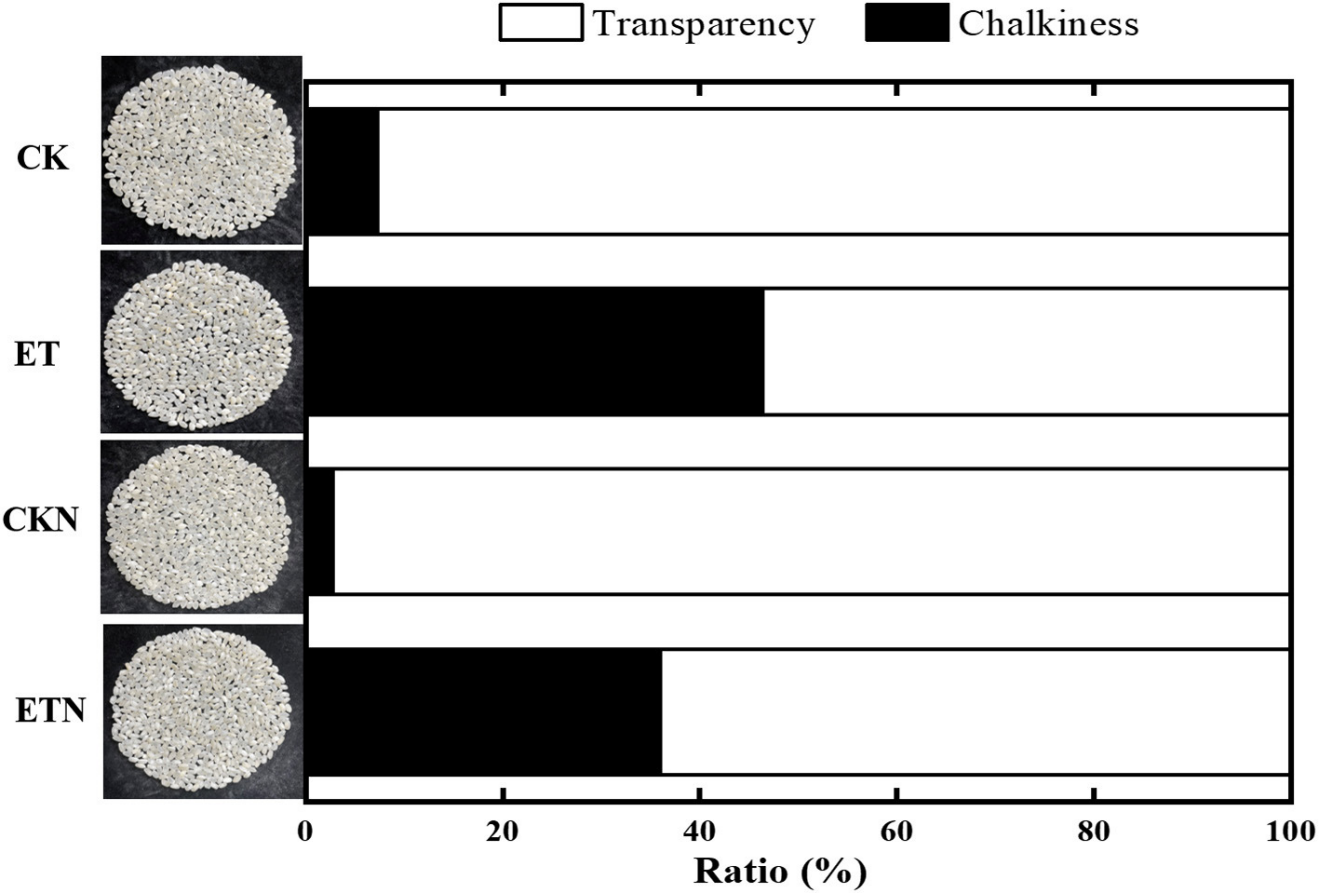
Effects of nitrogen fertilizer on grain chalkiness under elevated temperature. The rice plants were subjected to ambient temperature (CK), elevated temperature (ET), application of nitrogen fertilizer under ambient temperature (CKN), and application of nitrogen fertilizer under elevated temperature (ETN). Vertical bars represent mean ± SE (*n* = 3).

The results of actual paddy field warming on rice yield are basically consistent with previous studies on the impact of climate warming on rice yield. Compared with CK, ET had a negative impact on yield with a decrease of 23.72%. In particular, the increase in temperature has led to a significant decrease in the grain weight and the seed setting rate, which is also the main reason for the decrease in yield. Furthermore, in this study, the most prominent results of the effect of increasing temperature on the rice quality were the significantly increased chalky rice rate (197.67%), chalky area (104.62%), and chalkiness (532.92%). This result is also consistent with the results of our warming field trials carried out since 2012. Compared with the ET treatment, the application of nitrogen under ETN alleviated the loss of the grain weight and the seed setting rate to a certain extent and thus reduced the adverse effect of warming on yield (16.09%). Nitrogen fertilizer also had a significant effect on alleviating chalkiness with a decrease of 22.27%. However, nitrogen fertilizer had no significant effect on the chalky rate and chalk area (Tables 1, 2 and Figure 1). The milled rice rate and head rice rate were decreased by 3.93 and 8.33%, respectively, under elevated temperature, while nitrogen application increased the head rice rate by 4.34% at the significance level. The hot paste viscosity, peak viscosity, breakdown viscosity, and gelatinization temperature were increased by 14.43, 5.56, 23.72, and 5.39%, respectively, and the cool paste viscosity and setback viscosity were decreased by 8.01 and 175.45%, respectively, under elevated temperature. Nitrogen application could alleviate the changes of peak viscosity and breakdown viscosity but had no significant effect on other parameters (Table 2). Overall, the increase in temperature during the rice grain-filling stage has a certain negative effect on the yield components and quality indicators, while from the perspective of years of field trials, the application of nitrogen fertilizer has a positive regulatory role on the adverse effects of temperature increase and could be considered as a potential effective cultivation measure to cope with high-quality rice production under climate warming.

**Table 1:** Effects of warming and nitrogen fertilizer on yield and yield components in rice.

**Treatment**	**Spikelets per panicle (× 10^**4**^ hm^**−2**^)**	**Panicles per panicle**	**1,000 grain weight (g)**	**Seed setting rate (%)**	**Yield (t hm^**−2**^)**
CK	401.71a	96.10a	26.80a	95.35a	9.78a
ET	410.26a	86.02a	23.56d	89.70b	7.46c
CKN	418.80a	96.64a	26.03b	92.84a	9.77a
ETN	418.80a	90.77a	24.45c	93.24a	8.66b
T	ns	ns	^**^	^*^	^**^
N	ns	ns	ns	ns	ns
T*N	ns	ns	^**^	^**^	ns

*, ** *significant at 0.05 and 0.01 probability levels, respectively, ns means there is no significant difference. Values with different letters in each row represent for the significantly different with p < 0.05. T represents temperature, N represents nitrogen fertilizer, CK represents ambient temperature, ET represents elevated temperature, CKN represents application of nitrogen fertilizer under normal temperature, and ETN represents application of nitrogen fertilizer under elevated temperature*.

**Table 2:** Effects of nitrogen fertilizer on appearance, milling quality, and RVA characteristic parameters under elevated temperature in rice.

**Treatment**	**Chalkiness rate (%)**	**Chalkiness area (%)**	**Chalkiness (%)**	**Length (mm)**	**Width (mm)**	**Thickness (mm)**	**Milled rice rate (%)**	**Head rice rate (%)**	**Peak viscosity (cP)**	**Hot paste viscosity (cP)**	**Breakdown viscosity (cP)**	**Cool paste viscosity (cP)**	**Setback viscosity (cP)**	**Gelatinization temperature (^**°**^C)**
CK	28.78b	26.40b	7.32c	4.95a	2.93a	1.69ab	76.30a	73.60a	2931.50c	1500.50b	1431.00c	2573.00a	−358.50b	72.40b
ET	85.67a	54.02a	46.33a	4.73c	2.78b	1.71ab	73.3b	67.47c	3354.50a	1584.00a	1770.50a	2367.00b	−987.50c	76.30a
CKN	15.00c	20.12b	2.76d	4.96a	2.98a	1.67b	77.13a	73.83a	2804.50d	1478.50b	1326.00d	2521.00a	−283.50a	72.83b
ETN	82.67a	43.63a	36.01b	4.82b	2.77b	1.74a	73.83b	70.40b	3247.00b	1540.00ab	1707.00b	2293.50b	−953.50c	76.70a
T	^**^	^**^	^**^	^**^	^**^	^*^	^**^	^**^	^**^	^**^	^**^	^**^	^**^	^**^
N	^**^	^*^	^**^	^*^	ns	ns	^*^	^**^	^**^	ns	^**^	^*^	^**^	^*^
T*N	^*^	ns	^*^	ns	ns	ns	ns	^**^	ns	ns	ns	ns	ns	ns

*, ** *significant at 0.05 and 0.01 probability levels, respectively, ns means there is no significant difference. Values with different letters in each row represent for the significantly different with p < 0.05. T represents temperature, N represents nitrogen fertilizer, CK represents ambient temperature, ET represents elevated temperature, CKN represents application of nitrogen fertilizer under normal temperature, and ETN represents application of nitrogen fertilizer under elevated temperature*.

### Effects of Nitrogen Fertilizer on Endosperm Development and Grain Storage Proteins Under Elevated Temperature

#### Endosperm Development

**Figure 2:**
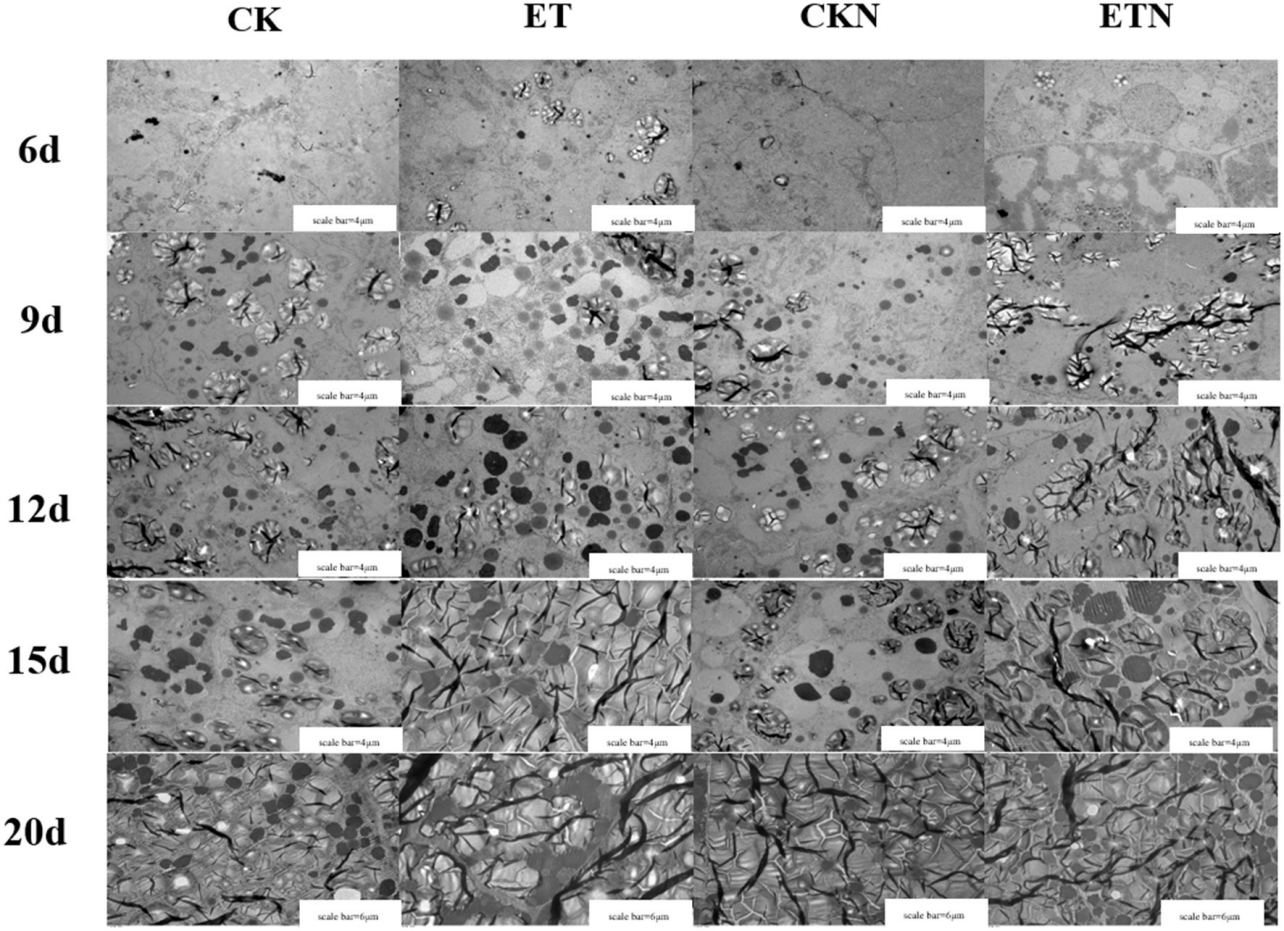
Effects of nitrogen fertilizer on dynamic changes of starch granules and protein bodies under elevated temperature during the grain-filling stage. The order of horizontal arrangement was CK, ET, application of CKN, and application of ETN, and the order of vertical column is 6, 9, 12, 15, and 20 days after anthesis (DAA). Scale bar = 4 μm at 6, 9, 12, and 15 DAA; scale bar = 6 μm at 20 DAA.

The effects of elevated temperature and nitrogen on the development of grain (superior pikelets) endosperm were investigated, and the results showed that there was no amyloplast found in CK and CKN treatments, while a small amount of amyloplasts and PB I and PB II were observed in ETN at 6 DAA. The PBs and amyloplasts in rice endosperm were increased rapidly, and the volume and quantity of PBII were increased significantly at 9 DAA. Results indicated that PBs were closely packed around amyloplasts under elevated temperature, while this phenomenon was not observed under normal temperature treatment in CK and CKN at 15 DAA. At 20 DAA, the endosperm of grain was basically mature, and the PBs of each treatment were closely packed among the amyloplasts. The PBs in ET were increased continuously and were tightly arranged in the endosperm and further connected and squeezed with the starch granules to fill the entire endosperm tissue. Compared with the ET treatment, the compression degree of amyloplasts and PBs under nitrogen treatment was relatively lower, indicating that the development of endosperms was slower than ET (Figure 2).

#### Dynamic Changes of the Protein Content

**Figure 3:**
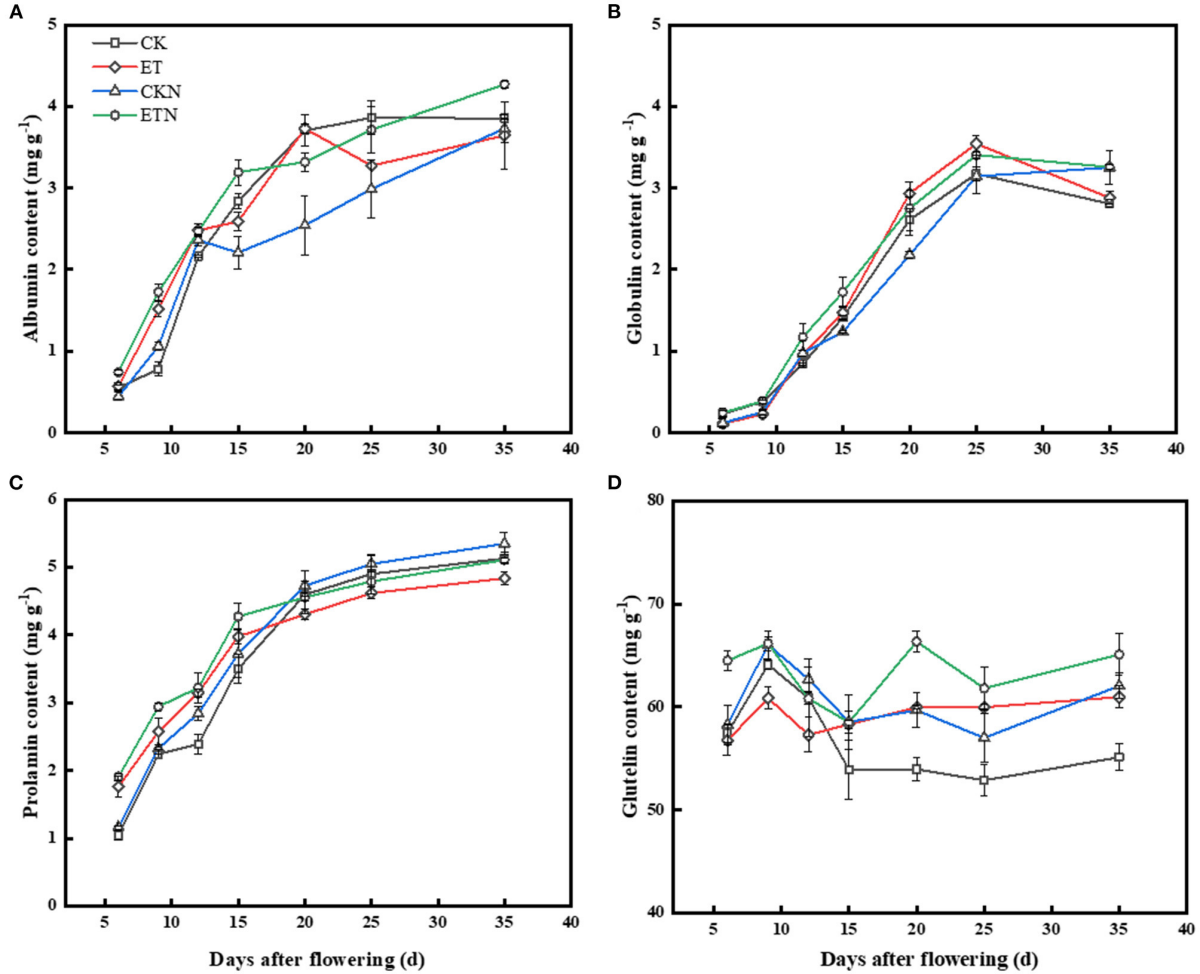
Effects of nitrogen fertilizer on grain dynamic content changes of protein components under elevated temperature during the grain-filling stage in rice. **(A)** Albumin, **(B)** globulin, **(C)** prolamin, and **(D)** glutelin. The rice plants were subjected to CK, ET, application of CKN, and application of ETN. Vertical bars represent mean ± SE (*n* = 3).

Results of grain storage protein components showed that when compared with CK, the albumin content of grains was increased at 6, 9, and 12 DAA under elevated temperature, and nitrogen application also had the same regulatory effect, while the difference in the albumin content of ET and ETN was not significant. Compared with CK, ET and ETN treatments increased the globulin content by 11.63 and 7.34%, respectively, at 25 DAA. Compared with CK, the prolamin content under the ET treatment increased after the initial stage of filling and then decreased, resulting in a relatively low prolamin content in the ET treatments. ETN significantly increased the prolamin content by 6.25% compared with ET during the whole grain-filling stage. Changes in the glutelin content indicated that at 15 DAA, elevated temperature obviously increased the glutelin content, while nitrogen application further increased its content. The glutelin content was increased by 10.61%, and the content of prolamin was decreased by 5.73% under elevated temperature at 35 DAA (Figure 3). Correlation results showed that the prolamin content was significantly and negatively correlated with chalkiness, hot paste viscosity, peak viscosity, breakdown viscosity, and gelatinization temperature, while it was positively related to head rice rate, amylose content, cool paste viscosity, and setback viscosity. The glutelin content was negatively associated with cool paste viscosity and was positively correlated with gelatinization temperature (Table 3).

**Table 3:** Correlation coefficients among grain quality and protein components in rice.

**Index**	**Albumin**	**Globulin**	**Prolamin**	**Glutelin**	**Amylose**	**Amylopectin**
Head rice rate	−0.11	0.09	0.94^**^	−0.25	0.94^**^	0.09
Chalkinesss	0.21	0.09	−0.92^**^	0.41	−0.94^**^	0.06
Amylose	−0.21	−0.01	0.90^**^	−0.40	1	0.20
Amylopectin	−0.40	0.27	−0.03	−0.16	0.20	1
Peak viscosity	0.18	0.11	−0.93^**^	0.37	−0.89^**^	0.04
Hot paste viscosity	0.06	0.07	−0.75^**^	0.17	−0.58^*^	0.00
Breakdown viscosity	0.20	0.16	−0.93^**^	0.41	−0.93^**^	0.05
Cool paste viscosity	−0.50	0.46	0.70^*^	−0.75^**^	0.86^**^	0.06
Setback viscosity	−0.31	0.24	0.90^**^	−0.53	0.93^**^	−0.01
Gelatinization temperature	0.40	0.34	−0.82^**^	0.65^*^	−0.86^**^	−0.01

*, ** *significant at 0.05 and 0.01 probability levels, respectively*.

### Effect of Nitrogen Fertilizer on the Protein Activities Related to Synthetase Under Warming Condition

**Figure 4:**
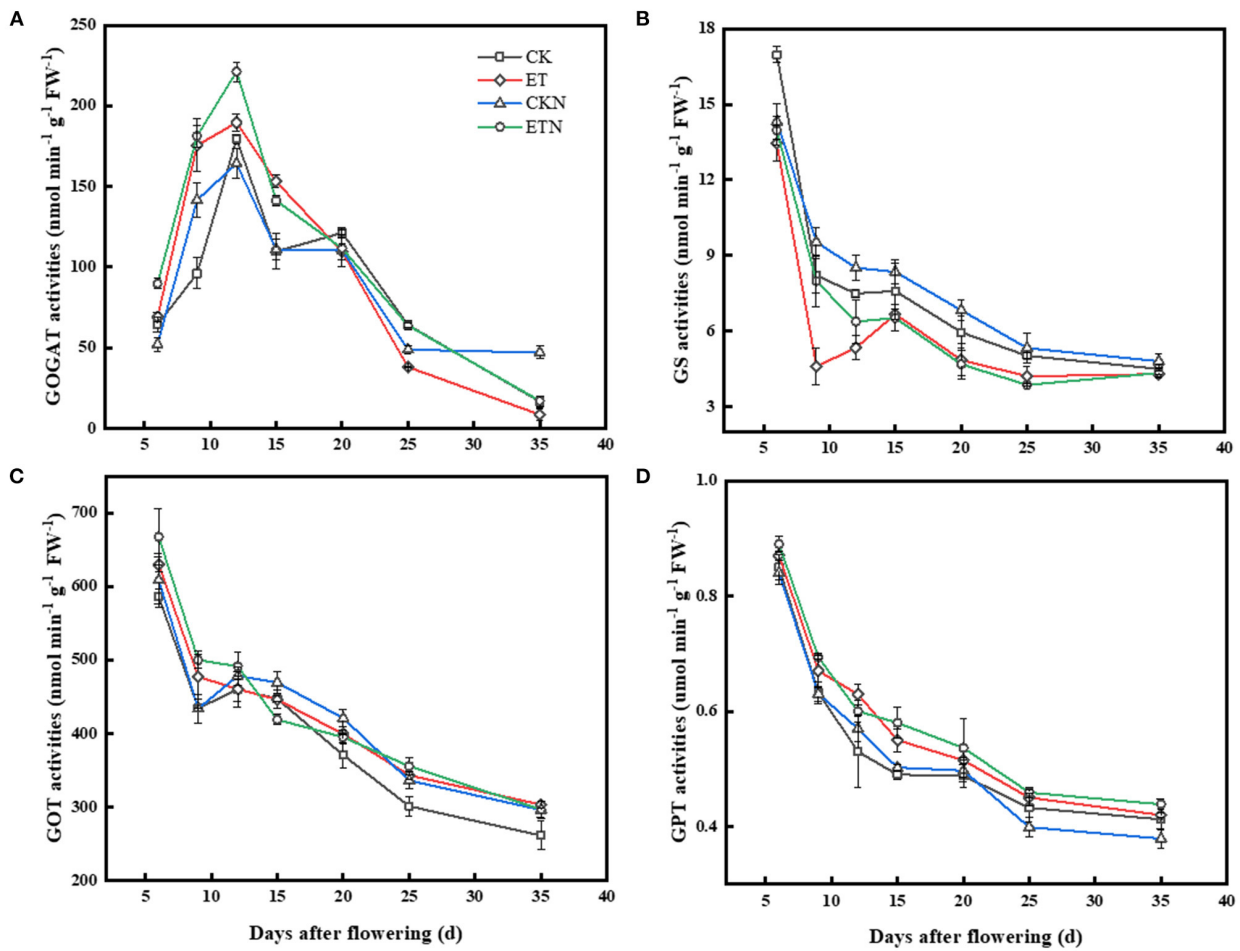
Effects of nitrogen fertilizer on grain nitrogen metabolism key enzyme activities under elevated temperature during the grain-filling stage in rice. **(A)** Glutamine synthetase, **(B)** glutamate synthetase, **(C)** glutamic oxaloacetic transaminase, and **(D)** glutamic pyruvic transaminase. The rice plants were subjected to CK, ET, application of CKN, and application of ETN. Vertical bars represent mean ± SE (*n* = 3).

The activity of GOGAT was relatively increased under elevated temperature before 20 DAA and was significantly increased by 39.48% at 15 DAA Compared with ET, the activity of GOGAT was increased in ETN during the whole grain-filling period. GS activity under the ET treatment was lower than that of the CK and was significantly decreased by 43.94 and 12.14% at 9 DAA and 15 DAA, respectively. Compared with the ET treatment, GS activity was significantly increased in the ETN treatment at 6 DAA, 9 DAA, and 12 DAA. However, the difference was not significant in the later stages in both ET and ETN treatments. During the whole grain-filling stage, increased temperature significantly increased the GOT activity, while nitrogen application under elevated temperature could further improve GOT activity until 12 DAA. Compared with CK, GPT was maintained higher activity in elevated temperature treatment, and nitrogen application under elevated temperature had similar regulatory effects on the activity of GPT (Figure 4).

### Regulatory Effects of Nitrogen Fertilizer on Main Regulatory Factors Related to Grain Protein Under Warming Conditions

**Figure 5:**
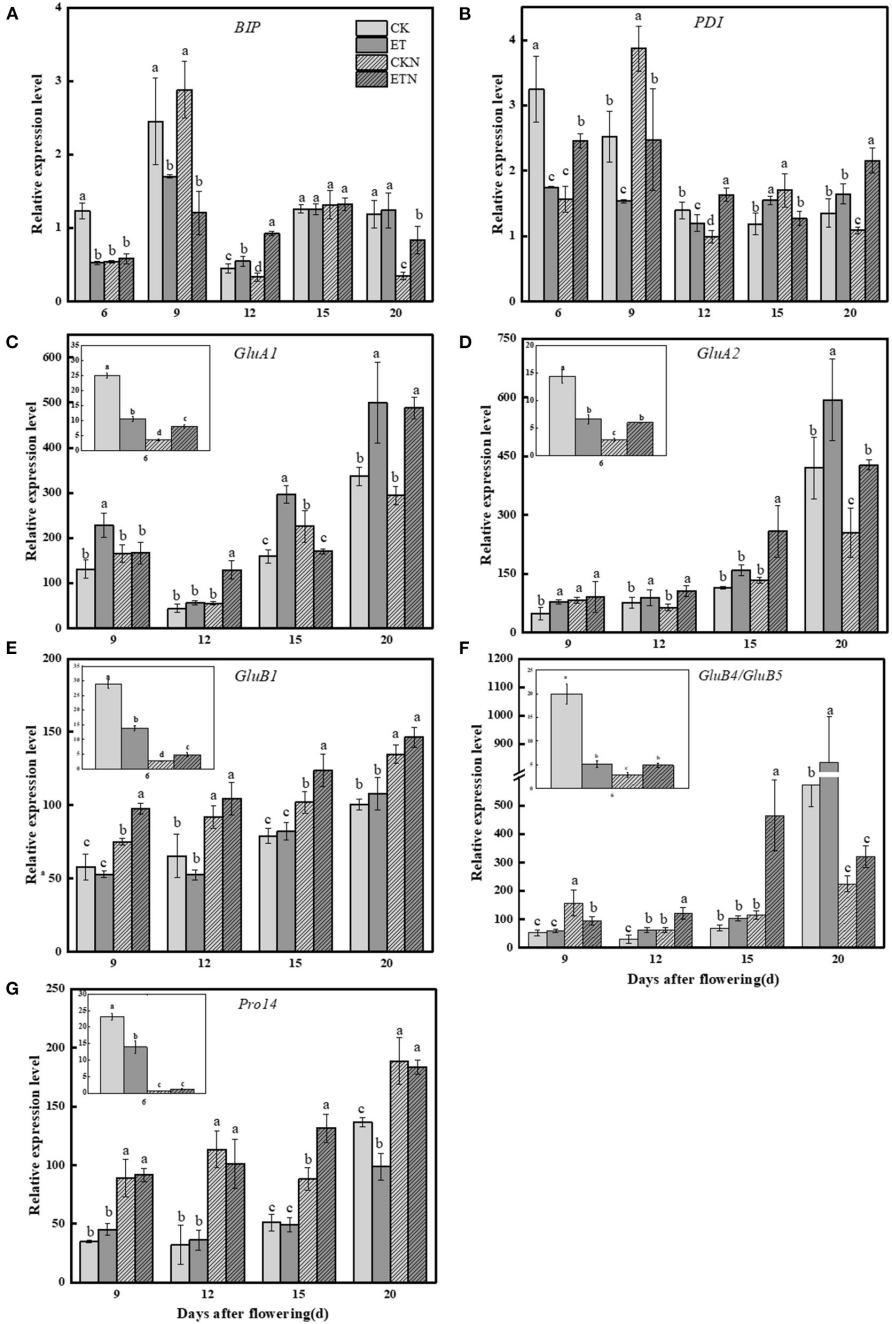
Effects of nitrogen fertilizer on grain nitrogen metabolism main regulatory factor genes under elevated temperature during the grain-filling stage in rice. **(A)** *BiP1*, **(B)** *PDIL1*, **(C)** *GluA1*, **(D)** *GluA2*, **(E)** *GluB1*, **(F)** *GluB5/GluB4*, and **(G)** *pro14*. The rice plants were subjected to CK, ET, application of CKN, and application of ETN. The small picture in the frame showed that the relative expression of each gene at 6 DAA. At the same grain-filling stage, different letters indicate significant differences among different treatments according to the Duncan's multiple range test (*P* < 0.05). Vertical bars represent mean ± SE (*n* = 3).

The regulatory effect of elevated temperature and nitrogen application on the currently known regulatory factors related to storage protein synthesis showed that increased temperature had a significant upregulating effect on both *GluA1* and *GluA2*. For example, compared with CK, the expressions of *GluA1* and *GluA2* were increased by 86.01 and 38.32%, respectively, at 15 DAA. The nitrogen application under elevated temperature decreased the relative expression of *GluA1* (except 12 DAA) but significantly increased the relative expression of *GluA2* (except 20 DAA). Under elevated temperature, the expression of *GluB1* at 6 DAA and 9 DAA was decreased and subsequently increased, while nitrogen increased the expression of *GluB1* under both ambient and elevated temperatures. The expression of *GluB5/GluB4* was significantly increased by 50.83% at 15 DAA under elevated temperature. Compared with CK, the relative expression of *pro14* was first increased and then decreased in ET. The application of nitrogen fertilizer effectively increased the relative expression of *pro14* in ETN treatment. The relative expression of *BiP1* was decreased by 30.63% at 9 DAA under elevated temperature. There was no significant difference in the relative expression of *BiP1* between ET and ETN treatments. The relative expression of *PDIL1* was decreased by 39.14% at 9 DAA and increased later by 30.29% at 15 DAA under elevated temperature (Figure 5).

## Discussion

### Free-air Temperature Enhancement (FATE) Facility

With the intensification of climate warming in recent years, the production of high-quality rice is facing new challenges. It is imperative to carry out studies on rice quality and cultivation strategies under this background. Since 2009, the FATE facilities have been introduced and continuously optimized to achieve the stable warming treatment in the actual paddy field with a limited influence on other environmental factors (Rehmani et al., 2014; Tang et al., 2019; Dou et al. 2017). Results of the warming effect of the FATE system indicated that when compared with CK, the average daytime temperature was increased by 2.78°C and the average night temperature was increased by 4.96°C in the ET treatment (Supplementary Figure 2). The warming effect of the FATE system was consistent with the IPCC prediction (global surface temperature was estimated to increase by 1.4–5.8°C by 2,100). Furthermore, the results indicated that the increase of night temperature was greater than daytime temperature, which is in line with the current global warming trend (IPCC, AR5, 2014). Therefore, based on the years of actual field test results, the FATE facility could be used to simulate the trend and characteristics of climate warming and that enables us to better assess the impact of temperature increase on rice quality and simultaneously evaluate the effects of nitrogen cultivation measures in coping with climate warming.

### Nitrogen Fertilizer Regulates Endosperm Development Under Elevated Temperature

When the temperature exceeds a certain limitation in the grain-filling stage, the grain transparency would be adversely affected (Dhatt et al., 2019). Decreased grain length or width could reduce the grain weight (Counce et al., 2005), and chalky grains generally exhibited inferior weight when compared with the CK (Wu et al., 2016; Nakata et al., 2017). Nitrogen application under increased temperature significantly reduced chalkiness, and it also decreased the grain length and weight caused by warming in this study, which is consistent with the abovementioned studies.

Studies had shown that the formation of chalkiness was closely associated with the grain-filling process. The increased temperature led to the obvious acceleration of early grain-filling rate and rapid decline in the mid-late period, resulting in poor filling of starch granules and PBs in the endosperm, which could further induce a large number of gaps, and thus forming chalkiness (Ito et al., 2009; Kobata et al., 2011). Both this study and our previous studies have shown that elevated temperature increased the rate of grain filling and significantly accelerated the development of endosperm (Figure 2). Previous studies on heat stress of rice showed that heat would induce a significant increase in protein storage vacuoles and less accumulation of storage protein, which eventually led to more gaps in protein storage vacuoles, thus resulting in chalkiness (Wada et al., 2019). Our results indicated that nitrogen application could effectively increase the protein accumulation in endosperm and that could further fill the gaps in protein storage vacuoles, thereby reducing chalkiness. Furthermore, the results also showed that the application of nitrogen fertilizer under elevated temperature could alleviate grain development, prolong the grain-filling period, and coordinate the development of amyloplasts and PBs (Figure 2), and it may be one of the main reasons for the decrease in chalkiness. However, Xi et al. (2021) showed that nitrogen application at the panicle differentiation stage promoted the formation of chalky grains. This may indicate that the mechanism of the formation of chalkiness is relatively complicated, and physiological factors, genetic factors, and ecological factors could co-modulate its formation. Therefore, conduct more in-depth studies, such as the use of transcriptome analysis and the construction of mutants, would be contributed to further revealing its mechanism.

### Nitrogen Fertilizer Affected the Grain Storage Protein Accumulation and Reduced the Formation of Grain Chalkiness Under Warming Condition

As the second major component, rice storage proteins are divided into albumin, globulin, prolamin, and glutelin according to the solubility (Shewry and Halford, 2002). Glutelin is the main component of grain storage protein, accounting for about 80% of seed storage proteins. The synthesis of glutelin begins with glutelin precursor subunits, which are then folded by a molecular chaperone, such as lumenal chaperon binding protein (BiP) and protein disulfide isomerase (PDI), transported into protein storage vacuoles, and formed mature acidic and basic subunits by splicing. Similar to the glutelin synthesis, prolamin consists of 10, 13, and 16 kDa protein subunits (Yamagata et al., 1982). The contents of albumin and globulin are relatively low and mainly distributed in the aleurone layer, pericarp, and embryo (Shewry and Halford, 2002). Storage protein plays an important role in the rice quality and is closely related to the formation of chalkiness (Liu et al., 2020). This study showed that the storage protein accumulated rapidly at 15–20 DAA (Figure 3), which was consistent with the study by Ashida et al. (2013), indicating that the storage protein is mainly accumulated in the middle grain-filling stage. Studies had shown that the high temperature decreased the starch content but this is conducive to the amino acids and the protein content accumulate in grains (Wang and Frei, 2011; Beckles and Thitisaksakul, 2014; Chun et al., 2015; Wang et al., 2019), and this feature was also obtained in the results of this study (Supplementary Tables 2–4). It is speculated that the increased temperature during the grain-filing stage promoted the activities of related enzymes, and the key regulators encoded the gene expressions, which jointly promoted the protein synthesis, and gradually accumulated the organic matter from source to sink organs, thus promoting the protein contents in rice grains (Lancien et al., 2000; Cao et al., 2017).

Results of the changes of protein components indicated that the total protein and glutelin contents were all increased, but prolamin was decreased under the condition of increased temperature (Supplementary Table 2). The influence of temperature on the glutelin content was significant, especially during 11–20 days after heading, which was consistent with the study conducted by Ashida et al. (2013).

The content of prolamin was significantly decreased under the warming condition, indicating that the decrease of the prolamin content may be related to the increase of chalkiness (Lin et al., 2010). This study showed that there was a significantly negative correlation between prolamin content and chalkiness, and Ishimaru et al. (2020) showed that the content of 13 kDa prolamin subunits in grains with more chalkiness was lower than that in transparent grains under field conditions. The transparency of grain was closely related to the physiological process of 13 kDa prolamin subunit synthesis under the high temperature. Previous studies have shown that transgenic lines with reduced 13 kDa gliadin exhibit a relatively transparent phenotype. Kawakatsu et al. (2010) constructed transgenic rice (SSP-less mutant), and the results showed that not only in the Glu-less mutant but also in the Pro-less mutant obtained no opaque phenotype. This may suggest that the formation of chalkiness is a complex process, which contains a multi-level of physiological and biochemical reactions. In our previous study, we stated that the balance changes of starch, protein, and other grain storage substances could be the possible reason for inducing the increased grain chalkiness. Therefore, it is, in fact, difficult to fully reveal the mechanism of the formation of grain chalk from only one aspect. Conducting more in-depth studies, such as the use of transcriptome analysis and the construction of mutants, would be contributed in further revealing its mechanism. Studies have shown that the protein and starch components of rice grain have a great influence on RVA characteristics (Champagne et al., 2009; Gu et al., 2015). Our results showed that the RVA parameters were changed under elevated temperature, and were also closely related to the changes of starch and protein contents of rice grains. Results showed that the protein content was significantly related to RVA characteristic parameters, and there was a significant correlation between amylose and RVA parameters. The amylose content was significantly positively associated with cool paste viscosity and setback viscosity and was negatively correlated with hot paste viscosity, peak viscosity, breakdown viscosity, and gelatinization temperature. This result was consistent with studies conducted by Yang et al. (2013), suggesting that the changes in the amylose content caused by warming would obviously change the cooking and eating quality of rice, which is also an important topic worthy of research under the background of climate warming.

### Regulatory Effects of Nitrogen Fertilizer on Main Regulatory Factors and Enzyme Activities Related to Nitrogen Metabolism Under Elevated Temperature

Carbon and nitrogen metabolism and its products are important factors that determine the rice quality (Hakata et al., 2012; Tang et al., 2018). Studies have identified the possible key metabolic steps related to starch in rice grains, as well as the role of key enzymes and regulatory factors in starch synthesis, such as granule bound starch synthase and starch branching enzyme (Yamakawa and Hakata, 2010; Yu and Wang, 2016). However, the relationship between nitrogen metabolites and the grain quality has not been given sufficient attention, and the role of activities of main nitrogen metabolism enzymes and regulatory factors is still unclear. More than 95% of inorganic nitrogen in plants is assimilated through the GS/GOGAT cycle (Hirel et al., 2001; Martin et al., 2006). Conversion of glutamic acid to other amino acids was achieved according to the catalysis of GOT and GPT, which provided substrates for protein synthesis. The elevated temperature was conducive to increase the key period activity of GOGAT (Liang et al., 2011). Studies had shown that the increase of GS and GOGAT enzyme activity can promote nitrogen metabolism, protein synthesis, and amino acid transformation (Lancien et al., 2000; Miflin and Habash, 2002). During the grain-filling process, the ammonia transfer process plays an important role in grain nitrogen metabolism. It was generally believed that GOT and GPT activities positively stimulated the protein content (Liang et al., 2011). In this study, the activities of GPT and GOT were increased significantly under increased temperature. Nitrogen application under elevated temperature could improve enzyme activities related to nitrogen metabolism, and the regulation of nitrogen fertilizer on enzyme activity was mainly during the early stage of grain filling (before 15DAA) (Figure 4). This may indicate that nitrogen and inorganic nitrogen, which provide substrates for protein synthesis, are assimilated into organic substances, such as proteins and nucleic acids, and participate in the GS/GOGAT cycle, thereby promoting protein synthesis (Yu et al., 2018; Huang et al., 2020).

Rice glutelin encoded by *GluA* and *GluB* subfamily is the important gene family for glutelin synthesis. Our results showed that the glutelin precursor was synthesized and accumulated after 6 DAA, and elevated temperature significantly increased the expression of *GluA1* and *GluA2*, while it decreased the expression of *GluB1* at the early grain-filling stage and increased its expression at the middle grain-filling stage, which was consistent with the changes of the glutelin content. The relative expression of *GluB1* and *GluB5/GluB4* (except 20 DAA) was evidently increased by the nitrogen application under the elevated temperature. The results showed that the expression of prolamin family gene*pro14* is more sensitive to the application of nitrogen during the whole filling stage under the elevated temperature. However, the changes in the expression of *pro14* induced by elevated temperature are not significant compared with normal temperature, which is somewhat different from the results of previous studies (Yamakawa et al., 2007; Cao et al., 2017). Furthermore, the tendency of *pro14* relative expression was consistent with the prolamin content changes under the elevated temperature and nitrogen fertilizer application, and it is speculated that *pro14* can be considered as the main factor in regulating the synthesis of prolamin under the background of warming and nitrogen, and more in-depth mechanism studies could be conducted.

In addition to family coding genes, molecular chaperones, namely, BiP and PDI, are also a kind of key regulators that regulate protein synthesis in rice grains. For example, after the precursor of gluten is transported to the cavity of the endoplasmic reticulum, it folds and assembles with the help of molecular chaperones in the cavity and generates disulfide bonds in the peptide chain (Takemoto et al., 2002; Kawakatsu and Takaiwa, 2010). However, conclusions on the expression pattern of molecular chaperones under the background of increased temperature or heat stress are still not consistent. In this study, elevated temperature significantly increased the relative expression of *PDIL1* in the middle grain-filling period, while *PDIL1* expression was decreased during the early grain-filling stage, and that may be the main reason for the decreased prolamin content detected in the early stage of grain filling. Expression of endoplasmic reticulum chaperone protein, *BiP1*, has been reported to be downregulated in chalky endosperm induced by high temperature (Ishimaru et al., 2009). Although, in this study, it was found that its expression was reduced in the early stage of grain filling, it is still not possible to fully infer the role of *BiP1* in the accumulation of storage proteins under the disposal of this study. Grain protein accumulation is a complex process involving multiple levels of the interaction among gene transcription, translation, protein folding, and degradation. Therefore, it is difficult to fully clarify the influence of temperature increase on protein synthesis and metabolism in rice grain, and more in-depth study, such as the use of transcriptome analysis, would be contributed to further reveal the mechanism.

## Conclusion

Our previous field study has shown that the application of nitrogen fertilizer can effectively regulate starch synthesis and reduce the appearance of grain chalkiness. In this study, the effects of elevated temperature and nitrogen fertilizer on grain metabolism were further elucidated. Results showed that nitrogen fertilizer could slow down the early grain development, prolong the grain-filling duration, and coordinate the development of PBs and starch bodies under the elevated temperature. Furthermore, nitrogen fertilizer affected the nitrogen metabolism enzyme and the key regulatory factors, which further regulated grain storage protein synthesis under elevated temperature, and this could induce the balance changes of grain storage substances and further regulate the grain quality. These findings would help us to better understand the impacts of global warming on rice quality and provide a theoretical basis for the establishment of high-quality rice cultivation approaches.

## Data Availability Statement

The datasets presented in this study can be found in online repositories. The names of the repository/repositories and accession number(s) can be found in the article/Supplementary Material.

## Author Contributions

ST and YD conceived the experiments. ST and XW designed the experiments. XW, KW, and TY performed the experimental study. XW wrote the manuscript. YZ, WL, and YS edited the manuscript. All authors read and approved the final manuscript.

## Conflict of Interest

The authors declare that the research was conducted in the absence of any commercial or financial relationships that could be construed as a potential conflict of interest.

## Publisher's Note

All claims expressed in this article are solely those of the authors and do not necessarily represent those of their affiliated organizations, or those of the publisher, the editors and the reviewers. Any product that may be evaluated in this article, or claim that may be made by its manufacturer, is not guaranteed or endorsed by the publisher.
