# Supplementary material for: Nitrogen Fertilizer Regulated Grain Storage Protein Synthesis and Reduced Chalkiness of Rice Under Actual Field Warming
Source: Front Plant Sci. 2021 Aug 30;12:715436. doi: 10.3389/fpls.2021.715436 (PMC8435852; doi:10.3389/fpls.2021.715436)
Supplement: Supplementary file 1 [file Data_Sheet_1.docx]

Supplementary Material

# Supplementary Figures and Tables

## Supplementary Figures


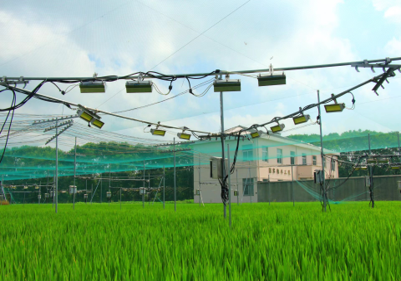


**Supplementary Figure 1.** The actual warming scenario in filed


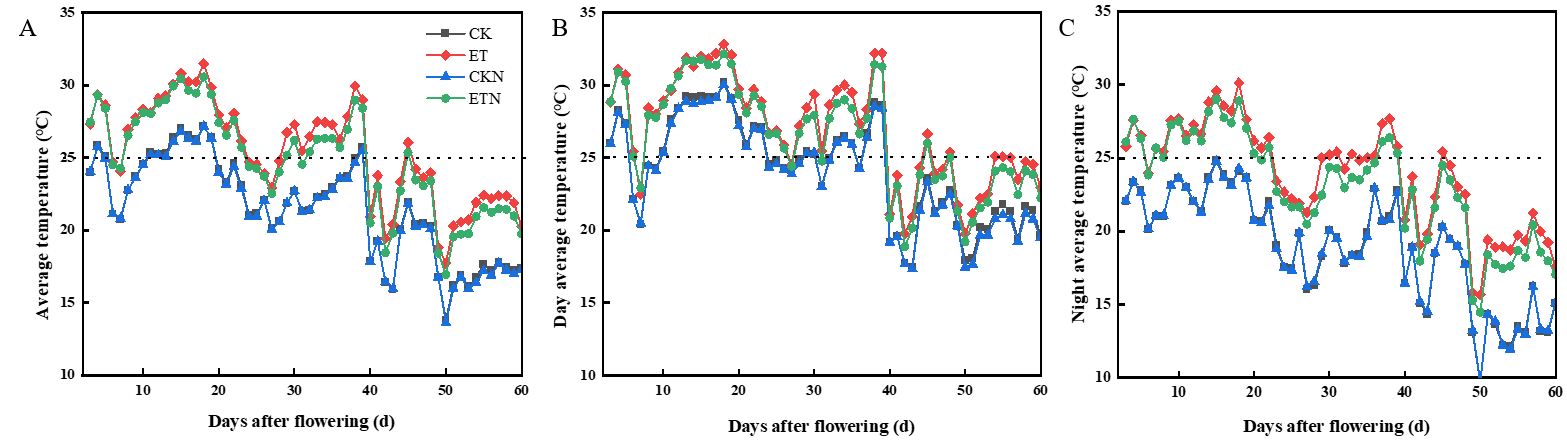


**Supplementary Figure 2.** Changes in temperature between different treatments during grain filling stage in rice. (A) average temperature, (B) daily average temperature, (C) night average temperature. The rice plants were subjected to ambient temperature (CK), elevated temperature (ET), application of nitrogen fertilizer under ambient temperature (CKN), application of nitrogen fertilizer under elevated temperature (ETN).

## Supplementary Tables

**Supplementary Table 1.** Primers used for quantitative real‐time RT‐PCR

| Gene | ID/Accession No | Forward primer | Reverse primer |
| --- | --- | --- | --- |
| *Actin* | 4349087 | CAATCGTGAGAAGATGACCC | GTCCATCAGGAAGCTCGTAGC |
| *Bip* | 4328075 | CTGTCGTGACTGTTCCTGC | GTTGGCTCATTGATGATCC |
| *PDI* | 4350002 | TGTCATCGCTAAGATGGATGC | AGCTCATCCTTGAGAGGCTCG |
| *Pro14* | Os05g0329100 | ACAACTCCAGCAGTTTGGTG | CAAGGGTGGTAATGGTACTG |
| *GluA2* | 4348563 | ATGATGGTGAAGTGCCGGTT | TCACGCCTGTATGCTTGAGG |
| *GluA3* | 4333164 | TCGGTTGCAAGCATTTGAGC | TCTGCCTGCAAGTGTGGATT |
| *GluB1* | [X54314](http://www.ncbi.nlm.nih.gov/nuccore/X54314) | GCCGTTCTGGAACGTCAATG | GACGAAGGCGTTAGCGTTTG |
| *GluB5/GluB4* | AK107238 | AATGGCGTTCTACGTCCAGG | TGCTTGCTCCCTCGATATGC |

**Supplementary Table 2.** Effects of warming and grain fertilizer on protein components of W3

| Treatment | Albumin  (%) | Globulin  (%) | Prolamin  (%) | Glutelin  (%) |
| --- | --- | --- | --- | --- |
| CK | 0.52c | 0.39bc | 0.73a | 6.06c |
| ET | 0.54bc | 0.37c | 0.64c | 6.67b |
| CKN | 0.56ab | 0.40b | 0.76a | 6.92b |
| ETN | 0.59a | 0.45a | 0.69b | 7.84a |
| T | ns | * | ** | ** |
| N | ** | ** | ** | ** |
| T*N | ns | ** | ns | ns |

Note: *, **, significant at 0.05 and 0.01 probability level respectively, ns means there is no significant difference. T represented temperature, N represented nitrogen fertilizer. CK represented ambient temperature, ET represented elevated temperature, CKN represented application of nitrogen fertilizer under normal temperature, ETN represented application of nitrogen fertilizer under elevated temperature

**Supplementary Table 3.** Changes of W3 starch components by warming and nitrogen fertilizer

| Treatment | Total starch  (%) | Amylose  (%) | Amylopectin  (%) | Amylopectin / Amylose |
| --- | --- | --- | --- | --- |
| CK | 70.41a | 17.74a | 52.67a | 2.97b |
| ET | 66.68b | 15.89c | 52.00ab | 3.15a |
| CKN | 67.83b | 17.63a | 50.20c | 2.85c |
| ETN | 68.51ab | 16.52b | 50.79bc | 3.20a |
| T | * | ** | ns | ** |
| N | ns | ns | ns | * |
| T*N | ** | ns | ** | ns |

Note: *, **, significant at 0.05 and 0.01 probability level respectively, ns means there is no significant difference. T represented temperature, N represented nitrogen fertilizer. CK represented ambient temperature, ET represented elevated temperature, CKN represented application of nitrogen fertilizer under normal temperature, ETN represented application of nitrogen fertilizer under elevated temperature

**Supplementary Table 4.** Effects of warming and nitrogen fertilizer on amino acid content and relative ratio of grains

| Amino acid | Content (mg g^-1^) | | | |  | | proportion (%) | | | | | | | |
| --- | --- | --- | --- | --- | --- | --- | --- | --- | --- | --- | --- | --- | --- | --- |
|  | CK | ET | CKN | ETN |  | | CK | | ET | | CKN | | ETN | |
| Met | 1.35b | 1.73a | 1.17c | 1.71a |  | 1.82b | | 2.19a | | 1.39c | | 1.85b | |  |
| Asp | 6.61c | 7.13b | 7.23b | 8.45a |  | 8.91ab | | 9.05ab | | 8.62b | | 9.11a | |  |
| Thr | 2.19b | 2.38ab | 2.30ab | 2.47a |  | 2.94ab | | 3.01a | | 2.74bc | | 2.67c | |  |
| Ser | 3.69b | 3.84b | 4.32a | 4.45a |  | 4.97ab | | 4.87b | | 5.15a | | 4.80b | |  |
| Glu | 14.28d | 15.20c | 16.32b | 17.98a |  | 19.24a | | 19.28a | | 19.46a | | 19.40a | |  |
| Gly | 3.08c | 3.21c | 3.36b | 3.82a |  | 4.15a | | 4.07a | | 4.13a | | 4.12a | |  |
| Ala | 4.57d | 4.91c | 5.44b | 5.95a |  | 6.15b | | 6.23aba | | 6.49a | | 6.42ab | |  |
| Cys | 0.47b | 0.62a | 0.71a | 0.76a |  | 0.64b | | 0.90a | | 0.75ab | | 0.82ab | |  |
| Val | 4.76c | 4.90c | 5.39b | 5.95a |  | 6.41a | | 6.20a | | 6.43a | | 6.42a | |  |
| Ile | 3.89c | 3.96c | 4.26b | 4.64a |  | 5.24a | | 5.02b | | 5.08ab | | 5.00b | |  |
| Leu | 6.46d | 6.91c | 7.45b | 8.10a |  | 8.70a | | 8.77a | | 8.88a | | 8.74a | |  |
| Lys | 3.26d | 3.35c | 3.50b | 3.98a |  | 4.39a | | 4.25bc | | 4.18c | | 4.29b | |  |
| Arg | 6.43c | 6.62c | 7.14b | 8.11a |  | 8.67ab | | 8.40b | | 8.51ab | | 8.75a | |  |
| Tyr | 4.22d | 4.41c | 4.80b | 5.12a |  | 5.69b | | 5.59b | | 5.75a | | 5.52b | |  |
| Phe | 4.64d | 5.11c | 5.41b | 6.01a |  | 6.25b | | 6.48a | | 6.45a | | 6.49a | |  |
| His | 2.17c | 2.17c | 2.40b | 2.67a |  | 2.92a | | 2.75c | | 2.86ab | | 2.88ab | |  |
| Pro | 2.15a | 2.41a | 2.65a | 2.55a |  | 2.90c | | 3.06b | | 3.43a | | 3.04b | |  |
| Total | 74.22 | 78.86 | 83.85 | 92.72 |  |  | |  | |  | |  | |  |

Note: Met: Methionine; Asp: aspartic acid; Thr: threonine; Ser: serine; Glu: glutamic acid; Gly: glycine; Ala: Alanine ; Cys: Cysteine; Val: valine; Ile: isoleucine; Leu: leucine ; Lys: lysine; Arg: arginine; Tyr:tyrosine; Phe: phenylalanine; His: histidine; Pro: Proline
